# Vitor Quality of Life Scale for the Elderly: evidence of validity and reliability

**DOI:** 10.1186/s40064-016-3130-4

**Published:** 2016-08-30

**Authors:** José Vitor da Silva, Makilim Nunes Baptista

**Affiliations:** 1 São Francisco University, Campus Itatiba, Itatiba, SP Brazil; 2 Wenceslau Braz School of Nursing, Rua João Faria Sobrinho, 61, apt. 301, Bairro Varginha, CEP: 37501-080 Itajubá, MG Brazil; 3 Sapucaí Valey University (UNIVAS), Pouso Alegre, MG Brazil

**Keywords:** Quality of life, Aged, Validation studies, Psychometrics

## Abstract

**Purpose:**

To assess validity and reliability of the Vitor Quality of Life Scale for the Elderly (VITOR QLSE).

**Methods:**

A sociodemographic questionnaire, a mental status questionnaire, the VITOR QLSE, the World Health Organization Quality of Life Instrument old module (WHOQOL-OLD), and the Baptista Depression Scale adult version (EBADEP-A) were administered to a non-probabilistic sample of 617 elderly persons living in Brazil. Exploratory factor analysis was performed to reduce the 70 items of the first version of the VITOR QLSE. Construct validity was then evaluated; the VITOR QLSE was tested against the WHOQOL-OLD and EBADEP-A. One hundred and ninety-two randomly selected participants completed the instrument again 7–15 days after the first interview, providing a test–retest reliability estimate.

**Results:**

Exploratory factor analysis reduced the 70 items to 48 items grouped into six domains: autonomy and psychological, environment, physical independence, family, health, and social domains. The total explained variance was 39.46 %. Cronbach’s alpha for overall reliability was 0.93 and ranged from 0.79 for health to 0.90 for physical independence. Pearson’s correlation coefficient (*r*) was 0.76 for test–retest reliability, and 0.56 and −0.57 for the correlation of VITOR QLSE scores with those of the WHOQOL-OLD and EBADEP-A, respectively.

**Conclusion:**

The final version of the VITOR QLSE has 48 items grouped into six domains and shows adequate validity and reliability. The rapid aging of the population and reduced number of instruments in the Latin America, and more specifically in Brazil, assessing quality of life in the elderly justify the development of a valid and reliable tool.

## Background

The rapid aging of the population in developing countries has become a current topic of interest, especially when it relates to the planning and allocation of public health resources for the care of the elderly (Jobim et al. 2010). The reduction of birth rates, increase in longevity, and declines in fertility and mortality have resulted in a sharp increase in the population aged 60 years and over. According to the World Health Organization (WHO), approximately two billion people in this age group will be living worldwide in 2050, and most of them in developing countries. Estimates indicate that 34 million people aged 80 years and over will be living in Brazil by 2025 (WHO 2001; Nunes et al. 2010). This phenomenon has been quite intense in Brazil; however, the society is unprepared to face such transition (Veras 2009). Statistics have shown a 60 % reduction in fertility rates over a 40-year period and a significant increase in life expectancy, tending to reach 77.08 years by 2025 (Brasil 2007). The number of elderly Brazilians rose from 2 million in 1950 to 15.4 million in 2002, accounting for a 700 % increase (Veras 2003). Projections have indicated that by 2025, Brazil will become the sixth country in the world with the largest elderly population (about 34 million people aged 80 years and over), which will correspond to approximately 15 % of the Brazilian population (Rezende et al. 2009; Nunes et al. 2010).

Aging is a natural process associated with physical, psychological, social and spiritual changes. Aging refers to a singular phenomenon involving endogenous factors and particular circumstances, not necessarily related to chronological age (Ciosak et al. 2011; Paschoal 2011). Thus, “aging” became a central theme in public policies, which value the necessity and importance of implementing measures to improve health and well-being in the elderly population. This boosts professionals from different health care disciplines to turn their attention to this phase of human development to find means to enhance odds of healthy aging and improve quality of life.

Quality of life is one of the most studied aspects regarding the elderly. It is a multidimensional and multifaceted construct that has been explored by researchers from different fields. It is expected that the better the quality of life, the greater the chances of a longer, healthier life with reduced risks of diseases and health conditions. The concept of quality of life includes objective and subjective factors, and therefore both the physical and psychological well-being should be considered (King et al. 1992; Kimura and Silva 2009). The concept of quality of life has been a topic of interest to researchers and the general public in recent decades. However, despite its importance, there is a lack of consensus for a single definition of the construct. Quality of life is a complex concept that incorporates several theoretical approaches and assessment methods (Kimura and Silva 2009). Quality of life is difficult to define because it is related to perceived life satisfaction or well-being, turning it into a subjective concept (Sousa et al. 2003; Mattos and Araújo 2009).

WHO defines quality of life as “the individuals’ perception of their position in life in the context of the culture and value systems in which they live and in relation to their goals, expectations, standards and concerns”. This definition includes six domains: physical health, psychological state, levels of independence, social relationships, environmental features, and spiritual concerns (WHOQOL Group 1995). Quality of life is a multidimensional term related to self-esteem and perceived well-being that involves several factors, including functional capacity, socioeconomic level, emotional status, social interaction, intellectual activity, self-care, family support, health status, cultural and ethical values, religiosity, lifestyle, job satisfaction or satisfaction in activities of daily living, living environment, perception of life events, behavior, happiness, and disease symptoms, among others (Santos et al. 2002; Velarde-Jurado and Avila-Figueroa 2002; Bowling et al. 2003; Sousa et al. 2003). Thus, the definition of quality of life changes from author to author; it is a subjective concept that depends on the sociocultural level, age group, and personal goals of the individual (Sousa et al. 2003).

According to Velandia-Mora (2002), for the elderly, quality of life results from interactions among different factors associated with the human existence, such as adequate housing, clothing, food, education, and autonomy. Each of these factors contribute in different ways to the ideal well-being, considering the aging process and adaptation of the individual to a changing biological and psychosocial environment, which occur in individualized and different forms. This adaptation influences the individual’s physical health, memory performance, functional ability, dependence, fear, abandonment, and death (Velandia-Mora 2002). An active social participation and engagement in joint activities perceived by the individual as beneficial have been considered as other significant elements to the quality of life of the elderly (Aragão et al. 2002). Krzemien (2001) indicated that the quality of life of the elderly is related to their history of significant interactions. In other words, if the elderly achieve recognition from significant social relations, this stage of life will be lived as an extension and continuation of a vital process; otherwise, it will be lived as a stage of functional decline and social isolation (Krzemien 2001). A wide variety of terms have been used to define quality of life, including a satisfactory life, subjective well-being, psychological well-being, personal development, and different conceptions of what makes “life good” (O’Shea 2001). Thus, quality of life should be investigated by asking the elderly what gives meaning to their lives within their cultural and value contexts, and how such contexts are related to their own goals in life (O’Shea 2001). Several factors affect quality of life of the elderly, including interpersonal relationships, physical and mental health state, material goods (e.g., housing, transportation, and income), access to health services, recreation, work, spirituality, community support, level of education, and environmental quality (e.g., safety and pollution), among others (Xavier et al. 2003; June 2004; Thomé et al. 2004; Vecchia et al. 2005).

Just as it is difficult to define quality of life, it is also difficult to measure it, because this concept can be influenced by cultural, ethical and religious values, as well as by personal values and perceptions (Thomé et al. 2004). For the assessment of quality of life in the elderly, biological, psychological and multiple sociocultural criteria should be adopted, because several factors are determinants or indicators of well-being in old age, such as longevity, biological health, mental health, life satisfaction, cognitive control, social competence, productivity, activity, cognitive efficiency, social status, income, continuity of family and occupational roles, and continuity of informal relationships with friends (Chikude et al. 2007).

Researchers from different fields search for valid, reproducible and reliable tools to assess quality of life in the elderly (Paixão and Reichenheim 2005), but the validity of such measures is difficult to be established, because there is no gold standard for comparison (Rufine et al. 2013). The World Health Organization Quality of Life Instrument old module (WHOQOL-OLD) is the only specific tool for assessing quality of life in the elderly available to date in Brazilian Portuguese (Power et al. 2005; Fleck et al. 2006).

The Quality of Life Index (QLI) (Ferrans and Powers 1985) is a generic measure of quality of life available in several languages, including Brazilian Portuguese. There are specific versions of the QLI for patients with cancer, lung issues, chronic fatigue syndrome, arthritis, diabetes, epilepsy, multiple sclerosis, spinal cord injury, stroke, home care patients, patients on dialysis, and those who had undergone kidney or liver transplantation (Ferrans and Powers 2008a, b). The generic version of the QLI was translated into Brazilian Portuguese, cross-culturally adapted, validated, and called Quality of Life Index Ferrans and Powers (IQVFP) in a previous study (Kimura 1999). The current generic version of the IQVFP is composed of 33 items grouped into four domains (Kimura and Silva 2009). Specific versions of the IQVFP have been developed to assess quality of life in people with wounds (Yamada and Santos 2009) and pregnant women (Fernandes and Vido 2009). The Vitor Quality of Life Scale for the Elderly (VITOR QLSE) was also developed based on the structure of the IQVFP and other methodological procedures (Ferrans 1996; Kimura 1999; Silva 2003; Gatti 2005). The initial VITOR QLSE includes domains representative of the quality of life of elderly people measured by 70 items that are simple and easy to understand, preventing fatigue or lack of motivation among respondents, especially in the case of debilitated, fragile elderly persons with low level of education.

Due to the increasing number of elderly persons, it is important to assess quality of life among this population, who face family, social, political, economic and health difficulties, because it is not enough to add years to life, but life to years (Paschoal 2011). However, there is a lack of specific tools on this topic in Brazil (Rufine et al. 2013).

The aim of this study was to assess construct validity and reliability of the VITOR QLSE, attempting to provide a reliable tool for assessing quality of life in the elderly.

## Methods

This quantitative study was approved by the Research Ethics Committee of the “Dr. José Antonio Garcia” School of Health Sciences, Sapucaí Valley University (UNIVÁS), Brazil (approval no. 957/08). All procedures were in accordance with the ethical standards of the 1964 Helsinki declaration and its later amendments. Written informed consent was obtained from all participants prior to their inclusion in the study.

### The participants

The target population consisted of elderly persons of both genders, living in urban areas of the cities of Itajubá, Piranguinho, Pouso Alegre, and Santa Rita do Sapucaí in the state of Minas Gerais (Brazil). The city maps of all participant cities were used to determine the study areas, which were chosen to be easily accessible to interviewers.

The convenience, non-probabilistic sample was composed of 617 elderly persons, a number proportional to the number of individuals living in the participating cities. Sample size was calculated to obtain stable factor solutions, using item-to-subject ratios. The minimum 1:5 item-to-subject ratio is necessary for testing the psychometric characteristics of a scale based on its internal structure (Pasquali 2010). However, the sample was almost nine times the initial number of items (n = 70) of the instrument.

Inclusion criteria were age ≥60 years, preserved cognitive and communication skills, being a resident in the participating cities, and agreeing to participate in the study.

The data were collected through direct structured interviews. The interviews were previously scheduled by personal contact or by phone and conducted at the participant’s home. The participants were recruited in public squares, workplaces, churches, and schools, among others. The interviews were performed from May to September 2013 by 10 properly trained investigators. Of the 647 elderly persons recruited to participate in the study, 20 individuals were not located after the interview was scheduled and 10 declined participation, for a final sample of 617 participants.

### The VITOR QLSE

The first version of the VITOR QLSE was developed through a qualitative analysis performed in a previous study (Silva 2003). A further review of the Brazilian literature was performed to identify important aspects related to the quality of life of the elderly, such as physical independence and environment (Neri 1993, 2008; Paschoal 2000, 2011; Lebrão and Duarte 2003; Sousa et al. 2003; Vecchia et al. 2005; Fleck et al. 2006; Mattos and Araújo 2009; Nunes et al. 2010). Focal group interviews were then conducted with 14 elderly persons selected from different subpopulations of seven different education levels (illiterate, incomplete and complete primary education, incomplete and complete high school education, and incomplete and complete college degree). The interviews were based on the techniques proposed by Gatti (2005) and coordinated by the first author of the study, who is a researcher experienced in conducting focus groups. This process resulted in the construction of the VITOR QLSE initially composed of 70 items grouped into eight domains: health/functioning (13 items); psychological/spiritual (10 items); social (10 items); family (9 items); citizenship (7 items); physical independence (5 items); autonomy (4 items); and environment (12 items), as shown in “Appendix”. The items are rated on a 5-point Likert scale ranging from “very unsatisfied” to “very satisfied” with higher scores indicating better quality of life. A pretest was conducted with an intentional sample of 30 elderly persons of the seven different education levels, who did not participate in the focus group. All participants in the pretest had no doubts about the items and content of the measure.

Thus, the 33-item IQVFP, which is the Brazilian version of the QLI-GV, underwent major modifications during the process of developing the VITOR QLSE.

More detailed information on the construction and pretesting of the VITOR QLSE will be published elsewhere. In this study, the validity and reproducibility of the instrument were assessed using exploratory factor analysis and test–retest analysis.

### Other instruments used in the study

Besides the VITOR QLSE, a sociodemographic questionnaire (Silva and Kimura 2002), a mental status questionnaire (Kahn et al. 1960), the Brazilian-Portuguese version of the WHOQOL-OLD (Fleck et al. 2006), and the Baptista Depression Scale - adult version (EBADEP-A) (Baptista and Cardoso 2012; Gomes and Baptista 2014) were used in the study.

A structured sociodemographic questionnaire (Silva and Kimura 2002) was used to assess sociodemographic characteristics (i.e., name, sex, age, race, education level, and religion, among others) and clinical characteristics (i.e., weight, height, medical history, medical conditions, and medications most used, among others) of the patients.

The mental status questionnaire (Kahn et al. 1960), containing 10 items assessing temporal and spatial orientation and memory, was used to test the cognitive status of patients recruited to participate in the study.

Construct validity using convergent validity analysis was tested by correlating VITOR QLSE scores with those of the WHOQOL-OLD and EBADEP-A.

The WHOQOL-OLD is the only specific measure of quality of life for use with older adults available to date in Brazilian Portuguese (Fleck et al. 2006), but the Brazilian version of the WHOQOL-OLD is not considered a gold standard. The WHOQOL-OLD consists in 24 items rated on a 5-point Likert-type scale and grouped into six subscales: sensory abilities, autonomy, past, present and future activities, social participation, death and dying, and intimacy. Subscale scores are combined into an overall score, which is transformed into a 0–100 scale, with higher scores representing better quality of life.

The ABADEP-A (Baptista and Cardoso 2012, Gomes and Baptista 2014) is a self-report measure of depressive symptoms in adults. It is composed of 45 items rated on a 4-point Likert-type scale and averaged into an overall score ranging from 0 to 135, with lower scores indicating less depressive symptomatology.

Test–retest was used to establish the stability-reliability of the VITOR QLSE. One hundred and ninety-two participants were randomly selected to complete the VITOR QLSE again 7 to 15 days after the first interview.

### Statistical analysis

Initially, parallel analysis was conducted using the FACTOR program version 10.3 (Lorenzo-Seva and Ferrando 2006). The estimation method of analysis was unweighted least squares (ULS) together with oblique factor rotation (Promin) based on a polychoric correlation matrix (Watkins 2006). Next, principal axis factoring and oblimin rotation were performed to determine whether the 70 items of the VITOR QLSE were suitable for factoring and how many domains the scale has. Free rotation of the entire sample was carried out for extraction of related factors. The factor-loading cutoff of 0.30 was used because it has been acknowledged as the minimum loading for indicating a meaningful contribution of a variable to a factor (Tabachnick and Fidell 2007), representing approximately 10 % of explained variance.

Cronbach’s alpha coefficient (α) was calculated to evaluate the internal consistency of the scale and its domains. Cronbach’s alpha indicates the degree to which a set of items measures a single unidimensional construct, determining the internal consistency or average correlation of items in a survey instrument and estimating its reliability. Cronbach’s alpha ranges from 0 to 1 and generally increases when the correlations between the items increase. The minimum acceptable value for alpha is 0.70; below this value the internal consistency of the scale is usually considered low. Alpha values between 0.80 and 0.90 are preferred (Nunnally 1978; Streiner 2003).

Pearson’s correlation coefficient (*r*) was used for test–retest analysis and to test the correlation of the VITOR QLSE scores against those of the WHOQOL-OLD and EBADEP-A. It indicates the extent of linearity that exists between the two variables. Its numerical value ranges from +1.0 to −1.0. It gives us an indication of the strength of relationship (Fulekar 2009). As a rule of thumb, the following guidelines on strength of relationship are often used, although many experts would somewhat disagree on the choice of boundaries: strong (*r* = −1.0 to −0.5 or 1.0 to 0.5); moderate (*r* = −0.5 to −0.3 or 0.3 to 0.5); weak (*r* = −0.3 to −0.1 or 0.1 to 0.3); none or very weak (*r* = −0.1 to 0.1).

The IBM SPSS Statistics for Windows, version 21.0 (IBM Corp., Armonk, NY, USA) was used for this analysis.

## Results

Exploratory factor analysis resulted in six non-random domains (KMO = 0.928; *P* < 0.001) with eigenvalues greater than 1.8, corresponding to 43.6 % of the explained variance, which may be considered acceptable. Four, five and six factors emerged from the analysis. A total of six factors were retained, which were considered to be the most appropriate for identifying a relevant number of items in each factor and allowing the exclusion of items with factor loadings ≤0.30 in more than one factor (Table 1). The explained variance for the VITOR QLSE domains was as follows: autonomy and psychological domain (ten items; 8.57 %), environment (ten items; 5.25 %), physical independence (six items; 7.40 %), family (seven items; 6.40 %), health (six items; 4.45 %), and social domain (nine items; 7.37 %) for a total explained variance of 39.46 %. Also, the six-factor solution was consistent with the parallel analysis. As a result, 22 items were removed, leaving a final version of the scale with 48 items. The six retained domains with their respective items are listed in Table 1. The 70 items of the VITOR QLSE before exploratory factor analysis are shown in “Appendix”. The items removed are indicated with an asterisk.

**Table 1:** Factor loadings for rotated items of the VITOR QLSE (n = 617)

Domain	Items	Factor loading
Autonomy and psychological	10. Possibility to live many years	0.485
	29. Leisure and fun activities	0.455
	31. Peace of mind and tranquility	0.422
	33. Achievement of personal goals	0.628
	34. Happiness	0.652
	35. General life	0.612
	36. Personal appearance	0.660
	37. Yourself	0.696
	38. Activities of daily living	0.582
	42. Type of life you are having	0.439
Environment	55. Safety in public roads	0.531
	62. Public road conditions	0.588
	63. Elevator, ramp and handrail in buildings or other places	0.486
	64. City buses	0.653
	65. Safety and comfort in city buses	0.720
	66. Public assistance received	0.562
	67. Special lines in banks, supermarkets and other places	0.768
	68. Amount of reserved lines in banks, supermarkets and other places	0.808
	69. Reserved parking spaces in banks, supermarkets and other places	0.700
	70. Amount of reserved parking spaces	0.745
Physical independence	08. Ability to take care of yourself	0.396
	43. Ability to move arms and legs	0.863
	44. Ability to walk back and forth	0.900
	45. Ability to take walks	0.794
	46. Ability to stand up	0.863
	47. Ability to get in and out of cars or buses	0.834
Family	12. Children	0.863
	13. Family happiness	0.513
	14. Sex life	0.443
	15. Husband/wife, boyfriend/girlfriend, companion	0.564
	48. How is the family	0.386
	49. Departure of children from home	0.800
	50. Professional choice of children	0.797
Health	01. Your health	0.397
	03. Pain intensity	0.801
	04. Pain intensity when performing activities of daily living	0.788
	05. Amount of medicine you take	0.675
	06. Effects of medicine you take	0.531
	21. Amount of concerns	0.449
Social	16. Friends	0.539
	18. Emotional support received from people	0.555
	22. Neighborhood	0.638
	23. House or apartment	0.485
	24. Your neighborhood	0.690
	40. Safety at home (house or apartment)	0.727
	41. Safety in your neighborhood	0.711
	59. Appreciation of other people	0.404
	61. Attention given by people	0.430

Pearson’s correlation coefficient among the VITOR QLSE domains and overall score (Table 2) ranged from weak (*r* = 0.14) to strong (*r* = 0.82) and showed statistical significance (*P* < 0.05). It is important to note that the correlation coefficients between the overall score and domains ranged between 0.56 and 0.82, which explains from 31 to 67 % of the covariance of the domains in relation to the overall quality of life assessed by the instrument.

**Table 2:** Correlation among VITOR QLSE domains and overall score (n = 617)

	Autonomy/psychological	Environment	Physical independence	Family	Health	Social	Overall VITOR QLSE
Autonomy/psychological	1	0.219	0.571	0.570	0.485	0.680	0.822
Environment	0.219	1	0.136	0.176	0.214	0.253	0.564
Physical independence	0.571	0.136	1	0.386	0.464	0.434	0.671
Family	0.570	0.176	0.386	1	0.362	0.537	0.697
Health	0.485	0.214	0.464	0.362	1	0.389	0.668
Social	0.680	0.253	0.434	0.537	0.389	1	0.767
Overall VITOR QLSE	0.822	0.564	0.671	0.697	0.668	0.767	1

Pearson’s correlation coefficient. Level of significance (*P* < 0.05)

The number of items per domain, Cronbach’s alpha coefficient, and Pearson’s correlation coefficient for the VITOR QLSE domains and overall score were as follows: autonomy and psychological (10 items; α = 0.89; *r* = 0.68), environment (10 items; α = 0.86; *r* = 0.73), physical independence (6 items; α = 0.90; *r* = 0.74), family (7 items; α = 0.81; *r* = 0.70), health (6 items; α = 0.79; *r* = 0.67), social (9 items; α = 0.86; *r* = 0.56), and overall score (48 items; α = 0.93; *r* = 0.76).

Various levels of correlation were obtained when comparing the VITOR QLSE overall score with scores of the WHOQOL-OLD and EBADEP-A due to the different characteristics of the subscales of these instruments. Pearson’s correlation coefficients between VITOR QLSE overall score and WHOQOL-OLD overall and subscale scores were as follows: sensory abilities (*r* = 0.24), autonomy (*r* = 0.35), past, present and future activities (*r* = 0.62), social participation (*r* = 0.64), death and dying (*r* = 0.10), intimacy (*r* = 0.47) and overall score (*r* = 0.56). A negative correlation was found between the VITOR QLSE and EBADEP-A overall scores (*r* = −0.57).

## Discussion

The exploratory factor analysis with oblimin rotation of 70 items resulted in the exclusion of 22 items. The six domains that were retained after the parallel analysis showed acceptable results, with factor loadings greater than 0.30 explaining 43.6 % of the variance, and correlation coefficients between the overall score and domains ranging from 0.56 to 0.82. In fact, lowest factor loading for an item was 0.39, greater than acceptable values (Tabachnick and Fidell 2007; Watkins 2006; Beavers et al. 2013). The correlation coefficients between overall quality of life and domains of the VITOR QLSE were greater than those reported by Pereira et al. (2006) for the Brazilian version of the WHOQOL-OLD.

The remaining 48 items were grouped into six domains. These domains are consistent with the literature or elderly reality, because they involve aspects related to the Elderly’s life that interfere in their quality of life. Paschoal (2011) noted that family, health aspects, physical independence, autonomy and religiousness, among other factors, interfere in the elderly’s perception of quality of life.

When comparing the VITOR QLSE domains with those of the IQVFP, which served as basis for the construction of the present tool, it was observed that the family, health, and social domains were maintained in the VITOR QLSE. Measures of quality of life for the elderly include specific domains, such as social, psychological and health factors, which largely impact the lives of the elderly (CFP 2008). The social recognition of the elderly is still very limited. Culturally speaking, the term “elderly” is associated with the concept of incapable (i.e., persons who have already fulfilled their goals and should give place to younger ones) and impaired individuals, with functional disabilities, cognitive problems, and unproductive. This population also faces social and professional losses due to retirement and reduced income, since in many cases the income decreases after retirement. They also experience friendship losses, widowhood and other losses throughout life (CFP 2008; Paschoal 2011).

From a psychological point of view, elderly persons want to live longer, participate in leisure activities, enjoy their free time, have some peace and quiet because they have already fulfilled their duties and responsibilities, and achieve their personal goals, which for a long time have been limited to a life plan. They may also want to search for happiness, improve their personal appearance, which is often affected by aging, be themselves, which is their right in a subjective point of view, and have a better life consistent with this stage they are living (CFP 2008).

Pereira et al. (2006) observed the impact of the physical domain on quality of life of the elderly worldwide, highlighting functional capacity and physical dependence as major factors affecting their lives. Ramos (2003) indicated that functional capacity is a new health paradigm for elderly people. Healthy aging becomes a multidimensional interaction among physical and mental health, independence in activities of daily living, social integration, and family support. The quality of life of the elderly has also been associated with economic independence (Sousa et al. 2003).

Pereira et al. (2006) also considered the physical environment, which is a domain of the VITOR QLSE, as the factor with the second highest influence in the global quality of life of the elderly. According to the WHO (WHO 2001), the physical environment in which elderly people live affects their level of independence. Usually, elderly people are physically and socially active if they can walk safely around their neighborhood, go to parks, or have access to a local public transportation. Elderly people who live in unsafe environments have lower chances of going out by themselves, and therefore are more likely to become isolated, depressed, have mobility problems, and bad physical condition, which are factors that influence their quality of life.

The relationship between the elderly and family members has to be evaluated in multigenerational households, since living together with the family may lead to positive and negative results. Family support has a positive effect on quality of life of elderly people, reducing isolation due to age-related impairment and functional dependence. On the other hand, intergenerational conflicts decrease self-esteem and affect the emotional status of the elderly, markedly reducing quality of life (Caldas 2003).

Health is a major influencing factor in quality of life, especially for the elderly. The presence of comorbidities or multiple pathologies may interfere with the elderly’s activities of daily living, functional capacity, independence, and autonomy. Awareness of finiteness and death may arise with a decrease in health, affecting their life satisfaction (d’Orsi et al. 2011; Paschoal 2011). Health professionals should emphasize the positive aspects of aging and show the importance of an adequate control of chronic diseases. When diseases are controlled, elderly people may enjoy the opportunities for recreation and entertainment at this stage of life.

The social domain is related to the social relationships of the elderly, including their opportunity to go shopping, talk on the phone, go for a walk, visit friends and family, and be recognized as individuals who have already fulfilled one stage of their lives. However, elderly persons are often not understood and may suffer prejudice because their social role may be devaluated by younger people.

The domains “autonomy” and “social participation” are present in both the WHOQOL-OLD and VITOR QLSE. Also, some items in the WHOQOL-OLD are similar to those in the VITOR QLSE, such as: “How satisfied are you with what you have achieved in your life?” “How satisfied are you with your activity level?” “How satisfied are you with your opportunities to seek other achievements in life?”

The VITOR QLSE domains and overall score had weak to strong correlation coefficients (range, 0.14–0.82), although all correlations were statistically significant (*P* < 0.05). High Cronbach’s alpha coefficients, ranging from 0.79 (health domain) to 0.93 (overall score), were obtained when assessing the internal consistency of the VITOR QLSE. Cronbach’s alpha ranging from 0.77 (family domain) to 0.93 (overall score) have been reported for the generic IQVFP scale (Ferrans and Powers 1985). For the WHOQOL-OLD with 24 items, Cronbach’s alpha coefficients range from 0.71 (autonomy subscale) to 0.88 (overall score) (Dancey and Reidy 2006). Yamada and Santos (2009) developed the Ferrans and Powers Quality of Life Index-Wound version (FPQLI-WV), which is a specific measure of quality of life for people with chronic wounds based on the IQVFP. The authors found Cronbach’s alpha coefficients of 0.90 for the overall score and 0.55, 0.65, 0.81, 0.88 for the family, socioeconomic, psychological/spiritual, and health/functioning subscales, respectively (Yamada and Santos 2009). Some of these values are lower than those estimated for the VITOR QLSE domains.

Test–retest reliability for the VITOR QLSE with a 7–15 days interval between tests indicated good temporal stability, with a correlation coefficient (*r*) of 0.76 for the overall score; all the correlations were statistically significant (*P* < 0.05). This is consistent with results reported for the QLI-GV with a test–retest interval of 2 weeks, showing a reliability coefficient of 0.87 among nursing students (n = 69) and 0.81 among a cohort (n = 20) of peritoneal dialysis patients (Ferrans and Powers 1985).

Fleck et al. (2006) found no significant differences in WHOQOL-OLD overall and subscale scores between test and retest performed at 7- to15-day intervals, with coefficients ranging from 0.58 (autonomy and intimacy subscale) to 0.82 (overall score), indicating that the instrument has good test–retest reliability; intraclass correlation coefficients for the overall and subscale scores also showed adequate values (Fleck et al. 2006). Leão (2012) evaluated the psychometric properties of the WHOQOL-OLD scale in 335 elderly people from Campina Grande (PB, Brazil). A six factor model was extracted by exploratory factor analysis. Two items were not loaded on any factor and, therefore, were excluded; the remaining items were loaded >0.3. The domains were reduced to three and the adjusted model performed better than the original one. Despite showing better values of Akaike information criterion (AIC), the root mean square error of approximation (RMSEA) remained above ideal (0.06) (Leão 2012). Thus, the WHOQOL-OLD presents psychometrical parameters acceptable but below the ideal for the elderly population of northeastern Brazil.

The WHOQOL-OLD is available in over 20 languages and used to assess subjective quality of life in the elderly. However, several studies are not representative because they used small samples to evaluate its psychometric properties. Conrad et al. (2014) tested the psychometric properties of this scale using a sample of 1133 elderly persons, 60 years old and over. Quality of life was assessed by the WHOQOL Short Form (WHOQOL-BREF), WHOQOL-OLD, and the Medical Outcomes Study 12-Item Short Form Health Survey (SF-12). In addition, the Geriatric Depression Scale (GDS), the Dementia Screening Test (Dem Test), and an Instrumental Activities of Daily Living (IADL) questionnaire were applied to evaluate depressive symptoms, cognitive capacity, and ability to perform activities of daily living (Conrad et al. 2014). The authors found Cronbach’s alpha of 0.85 in four subscales and 0.75 in two subscales of the WHOQOL-OLD. Internal consistency analysis indicated that all WHOQOL-OLD items significantly contributed to the measurement of their respective factors (Conrad et al. 2014). The internal consistency coefficient for the WHOQOL-OLD was similar to that found for the VITOR QLSE in the present study.

Liu et al. (2013) tested the psychometric properties of the Chinese version of the WHOQOL-OLD with 1050 elderly persons. The WHOQOL-OLD showed satisfactory reliability with Cronbach’s alpha of 0.711 for social participation and 0.842 for sensory capacity. All interclass correlation coefficients were above 0.7, indicating good test–retest reliability for the scale (Liu et al. 2013). The internal consistency of the Chinese version of the WHOQOL-OLD was higher compared to the VITOR QLSE, and both tools had temporal stability, although different statistical tests were used in each study.

Bowling and Stenner (2011) compared the psychometric properties of the Older People’s Quality of Life Questionnaire (OPQOL), the 19-item Control, Autonomy, Satisfaction, Pleasure (CASP-19), and the WHOQOL-OLD in three surveys conducted between 2007 and 2008 with older people living at home in Britan; however, only the OPQOL met criteria for internal consistency in the Ethnibus samples.

Caballero et al. (2013) validated the 13-item WHOQOL-AGE in a sample of 9987 elderly persons living in Spain, Finland and Poland. Velicer’s Minimum Average Partial (MAP) test divided the scale into two factors with a Cronbach’s alpha of 0.88 and 0.84 for factors 1 and 2, respectively. As in the VITOR QLSE, the results showed that quality of life is not a unidimensional construct. Although alpha values were high, they only exceeded internal consistency coefficients in the “family” and “health” domains.

The VITOR QLSE is a measure of quality of life specific for the elderly population whereas the IQVFP is a generic instrument, which can be administered to young and adult populations. The IQVPF is not structured with specific domains for the elderly population and its items do not assess aspects related to the aging process. Although the VITOR QLSE was developed based on the structure of the IQVFP, various items were carefully designed to assess the particular characteristics of the elderly population.

The correlation of the VITOR QLSE overall score with the WHOQOL-OLD overall and subscale scores and EBADEP-A overall score was close to the value considered excellent, according to the index by Prieto and Muniz (2000) for comparison of scales assessing the same construct. This indicates that there is convergent validity between the VITOR QLSE and the two instruments, even if some of the subscales of the instruments assess different aspects of the construct quality of life.

Convergent validity of WHOQOL-OLD was also tested against a measure of depression symptoms known as the Beck Depression Inventory (BDI) (Fleck et al. 2006). All the correlations between the BDI scores and the WHOQOL-OLD overall and subscale scores were statistically significant. A negative correlation between the two instruments indicates that the higher the levels of depressive symptoms, the lower are the subscale and overall scores on quality of life. The death and dying subscale had the lowest (*r* = −0222) and overall score had the highest correlation coefficient (*r* = −0.615) against the BDI; the other WHOQOL-OLD subscales showed satisfactory levels of correlation.

When compared with the WHOQOL-OLD, the VITOR QLSE has the advantage to cover factors commonly encountered in the daily life of the elderly that interfere with their quality of life, but that are not included in the WHOQOL-OLD, such as health, family, physical independence, and environment.

The development of a quality-of-life instrument that reflects the aspirations, desires, expectations, needs, fears, values and principles of the elderly is necessary due to the limited number of specific tools in Brazil and the peculiarities of this population. To accomplish this important task is to face and overcome the difficulties of a developing country, which has structural, organizational and political problems, and that suffers a rapid population aging process (Paschoal 2011).

### Study limitations

This study was conducted in southern Minas Gerais, Brazil. Further studies with different elderly populations in different socio-cultural contexts from different regions are necessary to obtain reference values for the Brazilian population, making it possible to compare studies. A non-probability design is less likely to produce representative samples and, therefore, probability sampling is recommended to be used in future studies. Additional longitudinal studies are needed to further test the performance of the VITOR QLSE. Further studies may investigate second-order factors for this instrument, perform item response analysis, and develop an abbreviated version of the VITOR QLSE, for applications in the frail elderly.

The Brazilian culture is very diverse due to the mixing of different ethnic groups in the Brazilian population, the large territory, and characteristics of each region of the country. The VITOR QLSE was culturally adapted to the southeastern region of Brazil, where the study was conducted. However, for the scale to be applied in other Brazilian regions, the evaluation of the items and a new cultural adaptation may be necessary, mainly because older people may retain many sociocultural aspects which will reflect in their speech and life style.

## Conclusions

The VITOR QLSE is composed of 48 items that are simple, short and easy to understand, measuring representative domains of the construct and preventing tiredness or demotivation of respondents, especially among the debilitated elderly or those with a low education level. The VITOR QLSE is a reliable measure of quality of life, showing internal consistency and temporal stability (test–retest reliability). Thus, it is recommended to measure quality of life among the elderly population in Brazil. The VITOR QLSE is an important tool for quality-of-life studies and may be used in research and clinical practice by multidisciplinary health professionals in the care of the elderly. Further studies are necessary to validate the VITOR QLSE in a Brazilian elderly population.

## Appendix

See Table 3.

**Table 3:** The 70-item VITOR Quality of Life Scale for the Elderly (VITOR QLSE) before exploratory factor analysis

How satisfied are you with…		Very dissatisfied	Dissatisfied	Neither satisfied nor dissatisfied	Satisfied	Very satisfied
1	Your health?	1	2	3	4	5
2*	The attention you pay to your own health?	1	2	3	4	5
3	Pain intensity?	1	2	3	4	5
4	Pain intensity when performing activities of daily living?	1	2	3	4	5
5	Amount of medicine you take?	1	2	3	4	5
6	The effects of the medicine you take?	1	2	3	4	5
7*	You memory?	1	2	3	4	5
8	Your ability to take care of yourself?	1	2	3	4	5
9*	Your ability to control your own life?	1	2	3	4	5
10	The possibility to live many years?	1	2	3	4	5
11*	The health of your family?	1	2	3	4	5
12	Your children?	1	2	3	4	5
13	Your family happiness?	1	2	3	4	5
14	Your sex life?	1	2	3	4	5
15	Your husband/wife, boyfriend/girlfriend, companion?	1	2	3	4	5
16	Your friends?	1	2	3	4	5
17*	The emotional support you receive from your family?	1	2	3	4	5
18	The emotional support you receive from others who are not family members?	1	2	3	4	5
19*	Your ability to be responsible for the family?	1	2	3	4	5
20*	How helpful are you?	1	2	3	4	5
21	Your mount of concerns?	1	2	3	4	5
22	The neighborhood?	1	2	3	4	5
23	Your house or apartment?	1	2	3	4	5
24	Your neighborhood?	1	2	3	4	5
25*	Your work (If you have any job with or without remuneration)?	1	2	3	4	5
26*	The fact that you do not have a job (due to retirement, unemployment or disability)?	1	2	3	4	5
27*	Years of education you completed?	1	2	3	4	5
28*	The way you manage your own money?	1	2	3	4	5
29	Your leisure and fun activities?	1	2	3	4	5
30*	Your possibility to have a happy future?	1	2	3	4	5
31	Your peace of mind and tranquility?	1	2	3	4	5
32*	Your faith in God?	1	2	3	4	5
33	Your achievement of personal goals?	1	2	3	4	5
34	Your happiness?	1	2	3	4	5
35	Your general life?	1	2	3	4	5
36	Your personal appearance?	1	2	3	4	5
37	Yourself?	1	2	3	4	5
38	Your activities of daily living?	1	2	3	4	5
39*	Your ability to make your own choices?	1	2	3	4	5
40	Safety at home (house or apartment)?	1	2	3	4	5
41	Safety in your neighborhood?	1	2	3	4	5
42	The type of life you are having?	1	2	3	4	5
43	Your ability to move your arms and your legs?	1	2	3	4	5
44	Your ability to walk back and forth?	1	2	3	4	5
45	Your ability to take walks?	1	2	3	4	5
46	Your ability to stand up?	1	2	3	4	5
47	Your ability to get in and out of cars or buses?	1	2	3	4	5
48	How is your family?	1	2	3	4	5
49	The departure of your children from home?	1	2	3	4	5
50	The professional choice of your children?	1	2	3	4	5
51*	Your religion?	1	2	3	4	5
52*	Your life experiences?	1	2	3	4	5
53*	Your current life?	1	2	3	4	5
54*	The amount of income received?	1	2	3	4	5
55	Safety in public roads?	1	2	3	4	5
56*	Your ability to make decisions?	1	2	3	4	5
57*	The health care received?	1	2	3	4	5
58*	Appreciation expressed by family members?	1	2	3	4	5
59	Appreciation expressed by other people?	1	2	3	4	5
60*	Life opportunities?	1	2	3	4	5
61	Attention given by people?	1	2	3	4	5
62	Public road conditions?	1	2	3	4	5
63	Availability of elevators, ramps and handrails in buildings or other places?	1	2	3	4	5
64	City buses?	1	2	3	4	5
65	Safety and comfort in city buses?	1	2	3	4	5
66	Public assistance received?	1	2	3	4	5
67	Reserved lines in banks, supermarkets and other places?	1	2	3	4	5
68	The number of reserved lines in banks, supermarkets and other places?	1	2	3	4	5
69	Reserved parking spaces in banks, supermarkets and other places?	1	2	3	4	5
70	Number of reserved parking spaces in banks, supermarkets and other places?	1	2	3	4	5

*** Items removed after exploratory factor analysis

## Abbreviations

*α* (alpha): Cronbach’s alpha coefficient
AIC: Akaike information criterion
CASP-19: 19-item Control, Autonomy, Satisfaction, Pleasure questionnaire
Dem Test: dementia screening test
FPQLI-W: Ferrans and Powers Quality of Life Index-Wound version
GDS: Geriatric Depression Scale
IADL: Instrumental Activities of Daily Living questionnaire
IQVFP: Quality of Life Index Ferrans and Powers - Brazilian version
MAP: Velicer’s minimum average partial test
OPQOL: Older People’s Quality of Life Questionnaire
QLI: Quality of Life Index
*r*: Pearson’s correlation coefficient
RMSEA: Root mean square error of approximation
SF-12: Medical Outcomes Study 12-Item Short Form Health Survey
UNIVÁS: Sapucaí Valley University (Universidade do Vale do Sapucaí)
VITOR QLSE: Vitor Quality of Life Scale for the Elderly
WHO: World Health Organization
WHOQOL-BREF: World Health Organization Quality of Life Short Form instrument
WHOQOL-OLD: World Health Organization Quality of Life Instrument Old module
